# Capacitorless Dynamic Random Access Memory with 2D Transistors by One-Step Transfer of van der Waals Dielectrics and Electrodes

**DOI:** 10.1021/acsnano.4c15750

**Published:** 2025-01-10

**Authors:** Jianmiao Guo, Ziyuan Lin, Xiangli Che, Cong Wang, Tianqing Wan, Jianmin Yan, Ye Zhu, Yang Chai

**Affiliations:** † Department of Applied Physics, The Hong Kong Polytechnic University, Kowloon, Hong Kong 999077, China; ‡ Joint Research Centre of Microelectronics, The Hong Kong Polytechnic University, Kowloon, Hong Kong 999077, China; § Research Institute for Smart Energy, The Hong Kong Polytechnic University, Kowloon, Hong Kong 999077, China

**Keywords:** capacitorless DRAM, 2D transistor, one-step transfer approach, vdW dielectric, h-BN tunneling layer

## Abstract

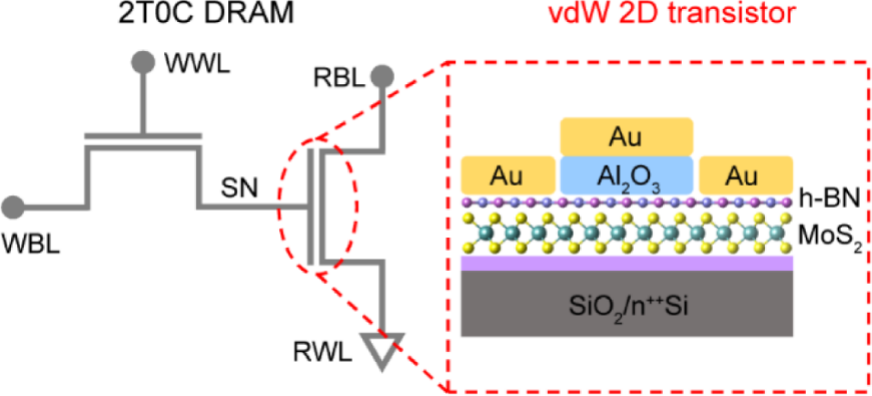

Dynamic random access memory (DRAM) has been a cornerstone of modern computing, but it faces challenges as technology scales down, particularly due to the mismatch between reduced storage capacitance and increasing OFF current. The capacitorless 2T0C DRAM architecture is recognized for its potential to offer superior area efficiency and reduced refresh rate requirements by eliminating the traditional capacitor. The exploration of two-dimensional (2D) materials further enhances scaling possibilities, though the absence of dangling bonds complicates the deposition of high-quality dielectrics. Here, we present a hexagonal boron nitride (h-BN)-assisted process for one-step transfer of van der Waals dielectrics and electrodes in 2D transistors with clean interfaces. The transferred aluminum oxide (Al_2_O_3_), formed by oxidizing aluminum (Al), exhibits exceptional flatness and uniformity, preserving the intrinsic properties of the 2D semiconductors without introducing doping effects. The MoS_2_ transistor exhibits an extremely low interface trap density of about 3 × 10^11^ cm^–2^ eV^–1^ and a leakage current density down to 10^–7^ A cm^–2^, which enables effective charge storage at the gate stack. This method allows for the simultaneous fabrication of two damage-free MoS_2_ transistors to form a capacitorless 2T0C DRAM cell, enhancing compatibility with 2D materials. The ultralow leakage current optimizes data retention and power efficiency. The fabricated 2T0C DRAM exhibits a rapid write speed of 20 ns, long data retention exceeding 1,000 s, and low energy consumption of approximately 0.2 fJ per write operation. Additionally, it demonstrates 3-bit storage capability and exceptional stability across numerous write/erase cycles.

## Introduction

Dynamic random access memory (DRAM) has long been the main memory in modern computing systems, but it now faces significant challenges as technology scales down. The mismatch between reduced storage capacitance and increasing OFF current presents obstacles in maintaining performance and efficiency.^1^ Capacitorless 2T0C DRAM architecture is recognized for its potential in DRAM technology because of superior area efficiency and reduced refresh rate requirements.^2−4^ By eliminating the traditional capacitor, capacitorless DRAM allows for the creation of higher-density memory arrays, enabling more data to be stored within a smaller physical footprint. Additionally, the exploration of two-dimensional (2D) materials offers exciting possibilities for further scaling down and achieving monolithic three-dimensional integration.^5−8^ These advancements could revolutionize DRAM technology, providing a pathway to overcome current limitations and meet the growing demands of modern computing applications, particularly in data-intensive fields like artificial intelligence.

Unfortunately, the inherent absence of dangling bonds of 2D materials poses a great challenge for the deposition of high-quality dielectrics on the surface of 2D semiconductors.^9−13^ Researchers have explored various techniques to deposit high-quality dielectric layers on 2D materials, such as plasma pretreatment and inducing defect sites.^14−17^ However, these methods often introduce trap states and damage the 2D lattices, degrading their physical properties. Using buffer layers has been considered to preserve the intrinsic properties of 2D materials, but these layers often lack stability and have low dielectric constants, affecting gate controllability.^18−22^ Aluminum oxide (Al_2_O_3_) can be easily formed by oxidation of aluminum (Al) with a low intrinsic defect density and consequently fewer defects at the interface between oxide and semiconductor, which minimizes the number of sites where charge carriers can be trapped. Additionally, with a wide bandgap of approximately 7 eV, Al_2_O_3_ offers excellent insulating properties and low leakage currents, which are essential for memory devices as they extend the retention of memory cells by preventing unintended charge loss.

In this work, we investigate a hexagonal boron nitride (h-BN)-assisted van der Waals (vdW) integration strategy for fabricating damage-free 2D transistors with pristine interfaces. The Al deposited on the monolayer h-BN is completely oxidized to Al_2_O_3_ through an annealing process and then stacked onto the 2D semiconducting channel via a one-step transfer process. This method separates high-energy deposition process from 2D materials, resulting in damage-free transistors with clean metal/2D and dielectric/2D interfaces. The resulting MoS_2_ transistors demonstrate intrinsic properties without doping effects, including a high ON/OFF ratio up to 10^8^, a low leakage current of ∼10^–7^ A cm^–2^, a small subthreshold swing (SS) of 84 mV dec^–1^, and extremely low interface trap density of about 3 × 10^11^ cm^–2^ eV^–1^. The monolayer h-BN tunneling layer also reduces the Schottky barrier height (SBH) to 98 meV. We successfully fabricated a 2T0C DRAM cell with two top-gate MoS_2_ transistors. The ultralow leakage current of the MoS_2_ transistor greatly enhances data retention, achieving a rapid write operation of 20 ns and data retention exceeding 1,000 s. This result demonstrates the potential of MoS_2_-based transistors in advancing DRAM technology.

## Results and Discussion

### One-Step Transfer of van der Waals Dielectrics and Electrodes Assisted by Monolayer h-BN

**Figure 1:**
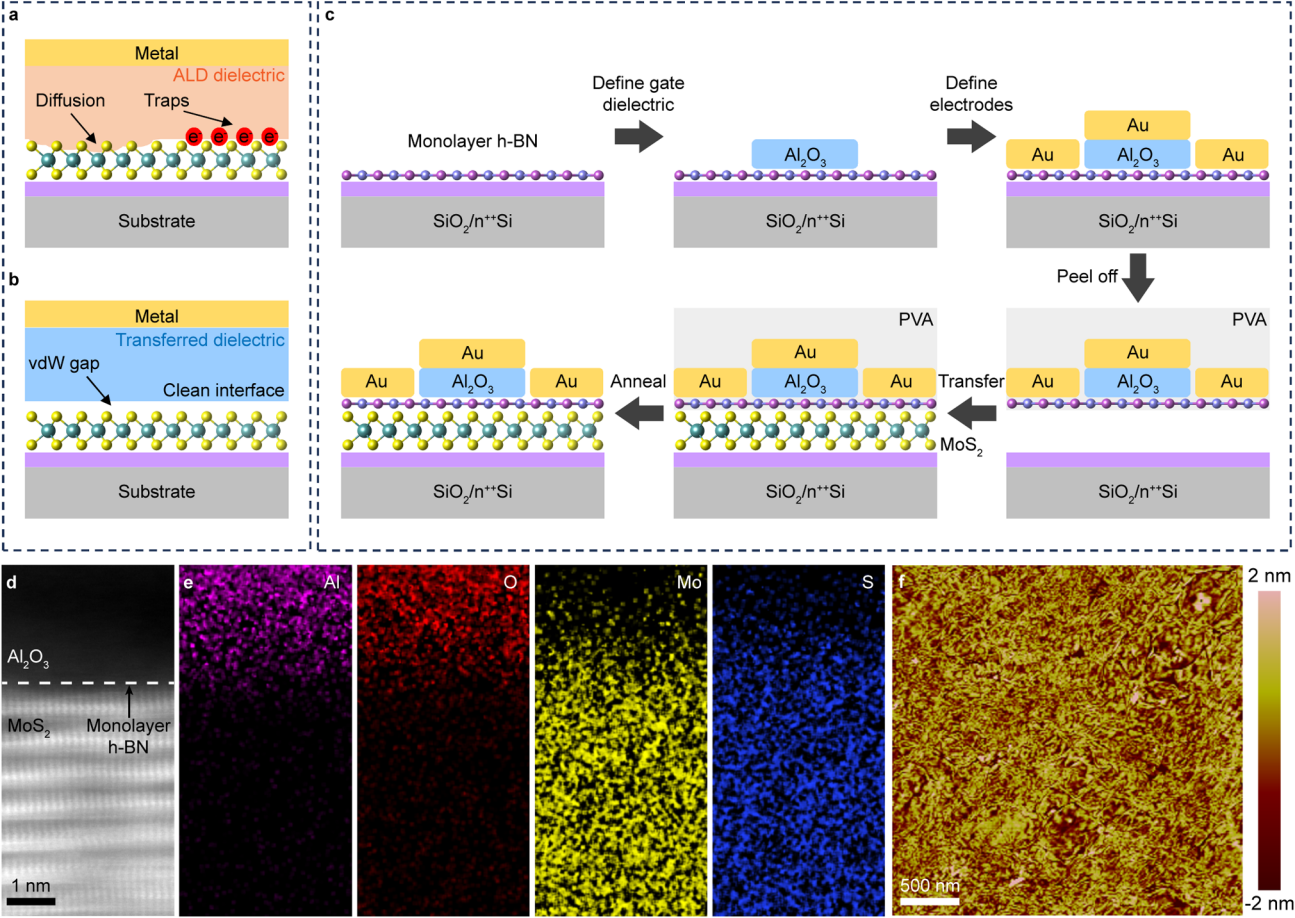
h-BN-assisted one-step dielectric/electrodes stack transfer process for damage-free top-gate transistors. (a) Cross-sectional view of top-gate MoS_2_ transistors with ALD dielectric. (b) Cross-sectional view of top-gate MoS_2_ transistors with transferred dielectric. (c) Schematic of the Al_2_O_3_ dielectric and Au electrodes transfer process for vdW integrated top-gate transistors. (d) High-resolution cross-section TEM image of the vdW integrated Al_2_O_3_/h-BN/MoS_2_ interfaces. (e) EDS mapping showing the elemental distribution of Al_2_O_3_/MoS_2_. (f) AFM image of the backside of peeled-off Au/Al_2_O_3_. The root-mean-square roughness is 0.8 nm.

A crucial challenge in fabricating 2D top-gate transistors is achieving a high-quality, reliable gate dielectric layer that can effectively control the electronic properties of the 2D material channel.^23−26^ Direct deposition of dielectric layers on the surface of 2D materials using atomic layer deposition (ALD) often results in the generation of interface traps and diffusion (Figure 1a).^18,27,28^ These defects can degrade the electrical performance of the device, leading to increased SS and hysteresis. Researchers have established vdW contact at the metal/semiconductor interfaces by transfer techniques, which results in a clean interface without chemical disorder and Fermi-level pining.^29−32^ We can also extend this strategy to transfer dielectric onto 2D semiconductors and create a clean dielectric/semiconductor interface (Figure 1b). Here, we adopted a monolayer h-BN as an interlayer for transferring dielectrics. The presence of h-BN weakens the strong adhesion between the dielectric/electrodes and SiO_2_ substrate, facilitating the ease of the transfer process. In addition, the insulating characteristics of h-BN suppresses current leakage.

Figure 1c schematically illustrates the transfer process of the dielectric/electrode stack. We selected Al_2_O_3_ as the gate dielectric due to its high dielectric constant, high breakdown voltage, and thermal stability.^33^ First, an Al film was patterned onto the monolayer h-BN using e-beam evaporation followed by a liftoff process. Then, the Al film was annealed in the air for oxidation and stress relaxation. The absence of a characteristic peak of Al at 72 eV in the X-ray photoelectron spectroscopy (XPS) spectrum is indicative of the complete oxidation of Al (Figure S1). Second, the source, drain, and gate electrodes were defined by standard photolithography and e-beam evaporation process at the same time. Thus, the complete device structure (dielectric and electrodes) was obtained on the SiO_2_ sacrificial substrate (Figure S2a). Third, the dielectric/electrode stack was peeled off from the substrate with the h-BN interlayer, because of the weak interaction between h-BN and substrate (Figure S2b). Next, the device stack was aligned and physically laminated on the exfoliated MoS_2_ flake by using a transfer platform. Finally, the poly(vinyl alcohol) (PVA) film was removed by deionization water. The fabricated device was annealed at 200 °C in vacuum to improve the contacts (Figure S2c,d).

This vdW integration method can create an atomically flat and clean dielectric/semiconductor interface, which can be confirmed by the cross-sectional TEM image (Figure 1d). The atomically flat and clean interface is crucial for damage-free transistors with minimum interface trap states. The energy dispersive spectrometer (EDS) mapping shows the elemental distributions of Al, O, Mo, and S in the corresponding area (Figure 1e). This elemental mapping confirms that the Al is uniformly oxidized and does not diffuse into MoS_2_. After peeling off the device stack from the sacrificial SiO_2_ substrate, we conducted an AFM examination of the backside of the dielectric region of the device stack. This analysis revealed an exceptionally flat surface characterized by a root-mean-square surface roughness of 0.8 nm (Figure 1f). This ultraflatness is attributed to the presence of the monolayer h-BN and the inherently smooth surface of the sacrificial substrate. The exceptional flatness of this surface suggests its potential to achieve intimate contact with MoS_2_, thereby enhancing the device performance.

### Electrical Characteristics of Damage-Free MoS_2_ Transistors

**Figure 2:**
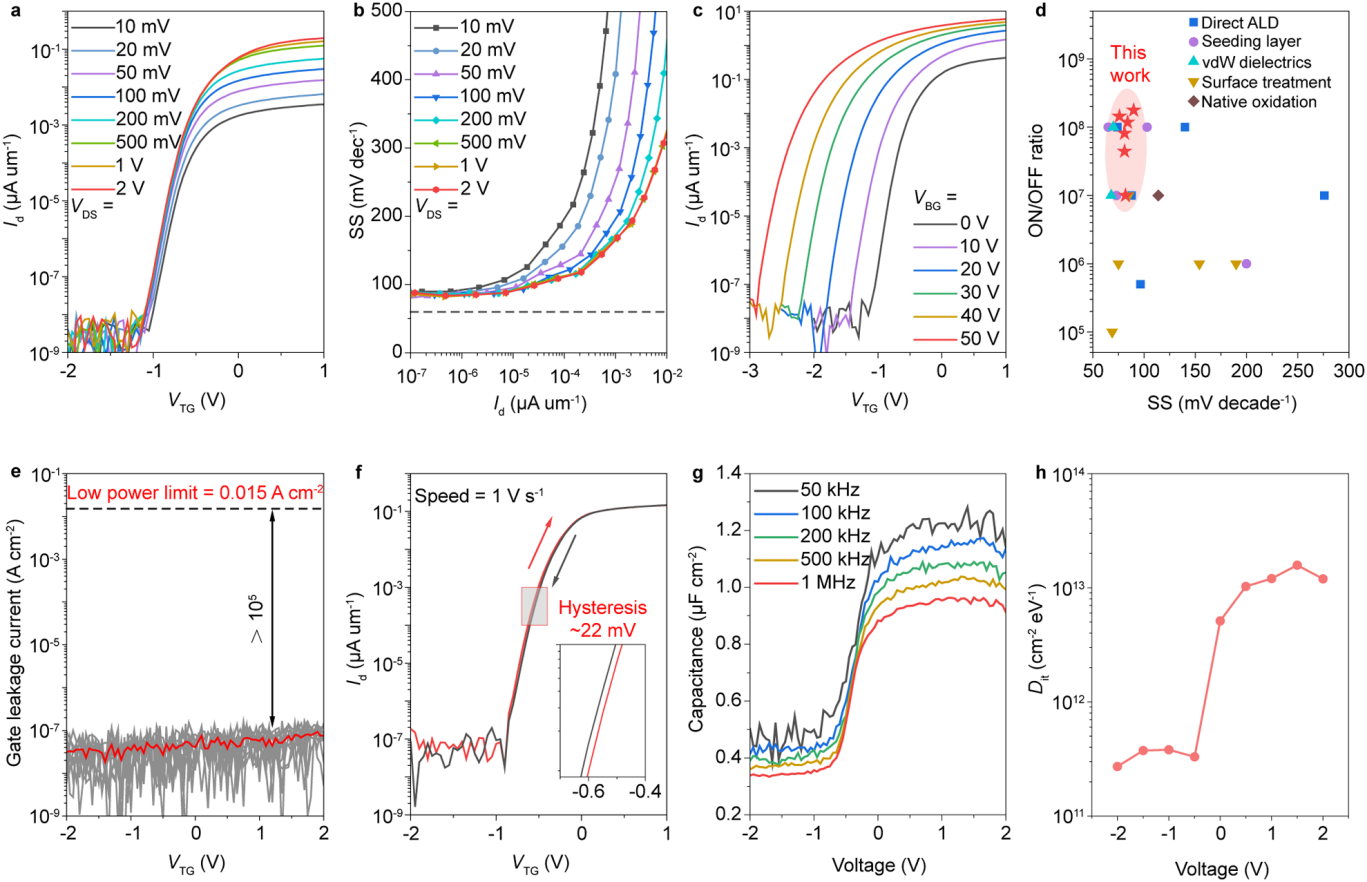
Electrical characteristics of damage-free top-gate MoS_2_ transistor. (a) Transfer curves of the vdW integrated damage-free top-gate transistor with different drain voltages. (b) Extracted SS at different channel currents of the top-gate MoS_2_ transistor. The black dashed line corresponds to the lower limit of 60 mV dec^–1^ at room temperature. (c) Transfer curves of the vdW integrated damage-free top-gate transistor under various back-gate voltages. (d) Comparison of the ON/OFF ratio and SS of our top-gate MoS_2_ transistor and other high-κ dielectric MoS_2_ transistor.^14−17,19−22,25,28,35−39^ The red stars represent measurements taken at different back gate voltages (0, 10, 20, 30, 40, and 50 V). (e) Gate leakage current density of the MoS_2_ transistor. The red line represents the average leakage current density of ten devices (gray lines). The black dashed line represents the low-power limit of 0.015 A cm^–2^. (f) Dual sweep transfer curve of the damage-free top-gate transistor at a speed of 1 V s^–1^. Back gate voltage and drain voltage are 0 and 1 V, respectively. The insert is a magnified view of the transfer curve in the gray box, indicating a hysteresis of ∼22 mV. (g) Capacitance–voltage (C–V) of the MoS_2_/h-BN/Al_2_O_3_ structure on a quartz substrate. (h) Extracted trap density as a function of top-gate voltage.

By employing this transfer methodology, the Al_2_O_3_ dielectric is physically laminated on the exfoliated MoS_2_ with a weak vdW force, thereby preserving the intrinsic properties of MoS_2_. The top-gate MoS_2_ transistor, featuring a transferred dielectric/electrodes stack, exhibits exceptional gate tunability. The transferred Al_2_O_3_-gated MoS_2_ transistor shows a high ON/OFF ratio of 10^7^ at a drain-source voltage of 1 V (Figure 2a). The OFF current is less than 0.1 pA, which indicates the high quality of thermally oxidized Al_2_O_3_. The device exhibits steep switching in the subthreshold region. Generally, SS quantifies the efficiency with which a transistor transitions from the OFF state to the ON state in response to the gate voltage and is given by the following equation:^13^
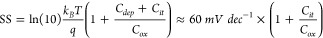
1where *k*_B_ is the Boltzmann’s constant, *T* is the absolute temperature, *q* is the elementary charge, *C*_it_ is the capacitance of interface traps, *C*_ox_ is the gate oxide capacitance, and *C*_dep_ is the depletion layer capacitance and is considered as zero in the subthreshold region due to complete depletion of the atomically thin channel. For an ideal 2D FET, the interface-trap density is negligible. This allows SS to reach its lowest limit of ∼60 mV dec^–1^ at room temperature, which represents the most efficient switching behavior for the transistor.

However, most top-gate 2D FETs have high interface trap densities caused by the dielectric deposition process, resulting in high SS values. Our device demonstrates a small SS of 84 mV dec^–1^ (Figure 2b), indicative of a low interface defect density. Without any back gate voltage, the device exhibits an ON/OFF ratio of 10^7^ and an ON-current of 0.4 μA μm^–1^ (Figure S3a). The relatively low ON-current can be attributed to the ungated regions that exist between the gate electrode and the contact electrodes. Increasing the back gate voltage enhances both the ON-current and the ON/OFF ratio, thereby confirming the dual-gate controllability of the device (Figures 2c and S3b). Figure 2d provides a comparative analysis of the ON/OFF ratio and SS of our device with recently reported high-κ dielectric-gated MoS_2_ transistor fabricated using various methods, such as ALD and seeding layer. The small SS value observed in our transferred Al_2_O_3_-gated MoS_2_ transistor can be attributed to the superior interface quality achieved through this transfer method, which notably avoids the introduction of impurities at the interface.

Leakage current is a crucial parameter in evaluating gate dielectrics within field-effect transistors. To assess the insulating properties of the transferred Al_2_O_3_, we measured the gate leakage current of our top-gate MoS_2_ transistor (Figure 2e). The observed ultralow gate leakage current density of <10^–7^ A cm^–2^ is well below the low-power limit for complementary metal-oxide-semiconductor devices, significantly reducing the static power consumption of our devices. Figure 2f shows the dual sweep transfer curve of the fabricated top-gate MoS_2_ transistor, indicating a small hysteresis of 22 mV. The small hysteresis can also be ascribed to the superior interface quality. Maintaining high-quality interfaces is of paramount importance for achieving high performance in 2D FETs. To quantitatively analyze the interface trap density *D*_it_, high-low frequency capacitance–voltage (C–V) measurement was performed (Figure 2g). *D*_it_ can be calculated by the following equations:
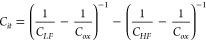
2
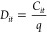
3where *C*_it_ is the capacitance of interface traps, and *C*_LF_ and *C*_HF_ are the capacitances measured at low and high frequencies, respectively. The extracted *D*_it_ shows an interface trap density of about 3 × 10^11^ cm^–2^ eV^-1^ in the accumulation region (Figure 2h). The relatively low interface trap density, compared to ALD dielectrics (which are usually greater than 2 × 10^12^ cm^–2^ eV^-1^),^34−36^ corroborates the electronically pristine interfaces achieved through the damage-free vdW fabrication approach.

### Reducing Contact Resistance with Monolayer h-BN as a Tunneling Layer

**Figure 3:**
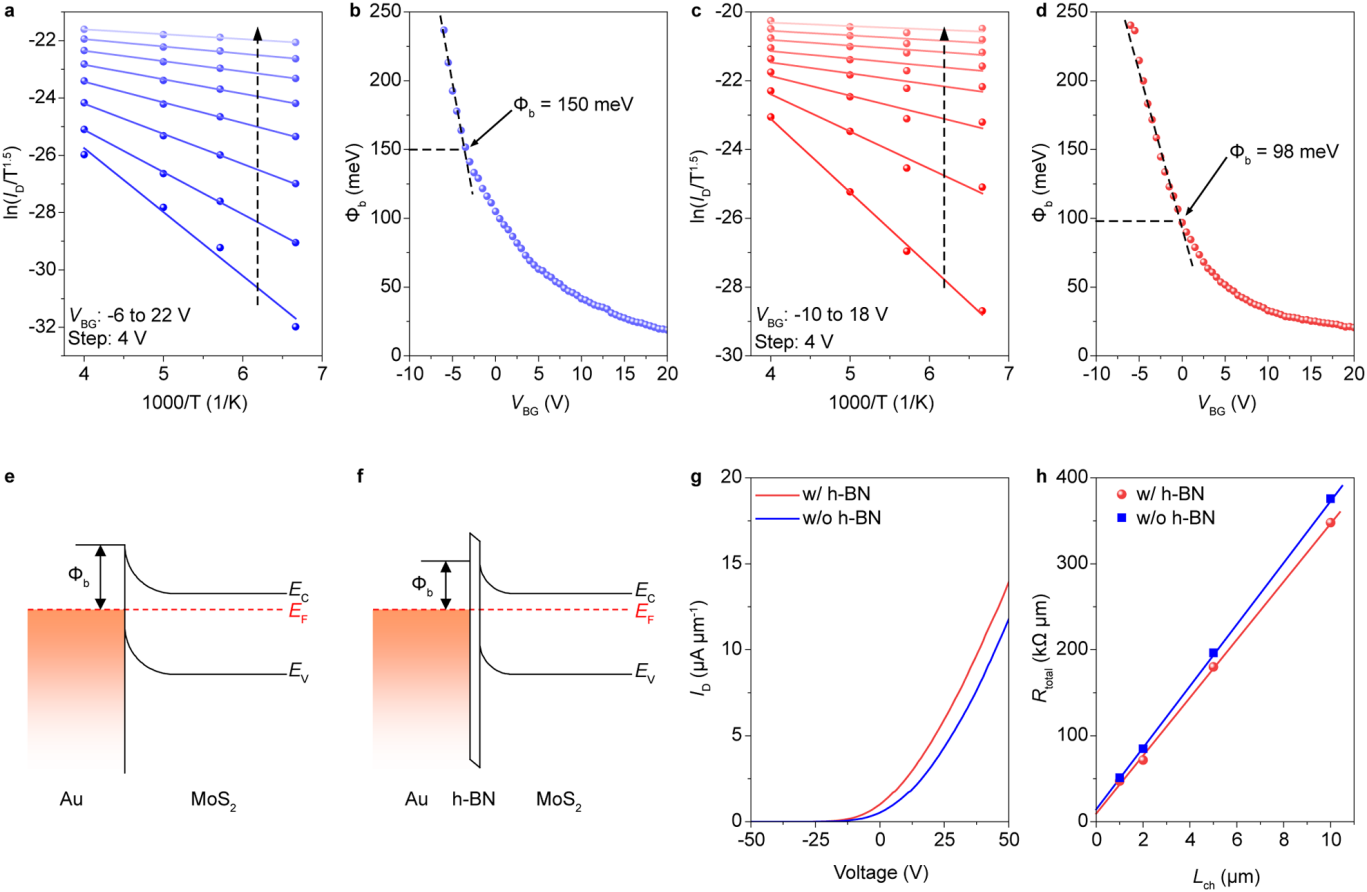
Monolayer h-BN serves as a tunneling layer to reduce the SBH and contact resistance. (a) The Arrhenius plot of back-gated MoS_2_ transistor with transfer Au electrodes at *V*_DS_ = 1 V. (b) The extracted energy barrier height at different back gate voltages. (c) The Arrhenius plots of back-gated MoS_2_ transistor with transfer h-BN/Au electrodes at *V*_DS_ = 1 V. (d) The extracted energy barrier height at different back gate voltages. (e) Band diagram for Au contact, MIGS results in a high SBH. (f) Band diagram for h-BN/Au contact, the Schottky barrier is reduced by minimizing the penetration of MIGS. (g) Transfer curves of MoS_2_ transistors with Au or h-BN/Au contacts. (h) Comparison of the contact resistances of the MoS_2_ transistors with/without the h-BN tunneling layer.

When a 2D semiconductor contacts a three-dimensional (3D) metal, the Fermi level at the interface is typically pinned at a specific energy level within the bandgap of the 2D semiconductor. This pinning effect remains relatively invariant with respect to the work function of the contact metal, resulting in a high SBH and elevated contact resistance. To mitigate the effects of Fermi level pinning at the interface between a 2D semiconductor and a 3D metal, researchers demonstrated an approach involving the insertion of an ultrathin tunneling layer between them.^40^ Therefore, one reason for selecting monolayer h-BN to facilitate the transfer of the device stack is its capability to function as a tunneling layer, which reduces the SBH and consequently lowers the contact resistance. To analyze the SBHs of Au and h-BN/Au contacts, back-gated MoS_2_ transistors with/without the h-BN tunneling layer were fabricated on the SiO_2_/n^++^Si substrate. Transfer curves of the MoS_2_ transistors at different temperatures are shown in Figure S4. According to the thermionic emission theory, the current of the transistor (*I*_D_) can be written as
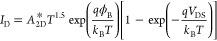
4where *A*_2D_^*^is the 2D equivalent Richardson constant, *T* is the absolute temperature, Φ_B_ is effective barrier height, *k*_B_ is the Boltzmann constant, *q* is the electronic charge and *V*_DS_ is the drain to source voltage. The thermionic emission current dominates the current in the transistor when the back gate voltage (*V*_BG_) is lower than the flat-band voltage (*V*_FB_). However, when *V*_GS_ exceeds *V*_FB_, a significant thermally assisted tunneling current emerges, leading to nonlinear behavior in Φ_B_-*V*_GS_ plot. At *V*_GS_ = *V*_FB_, SBH can be determined by observing the transition point where the relationship between barrier height and *V*_GS_ changes from linear to nonlinear. To obtain the barrier heights, the values of ln(*I*_D_/*T*^1.5^) are plotted at fixed *V*_DS_ of 1 V with different gate voltages (Figure 3a and 3c). The slopes extracted from the Arrhenius plot exhibit a linear relationship with *V*_DS_, as shown in Figure S4c,d. The y-intercept of Figure S4c,d, denoted by *S*_0_, represents the extrapolated slope value at *V*_DS_ = 0 V. The energy barrier height is then determined by Φ_b_ = −1000*S*_0_*k*_B_/q. The resulting energy barrier heights at various gate voltages are plotted in Figure 3b,d. For comparison, the SBHs are extracted to be 150 meV without the h-BN tunneling layer and 98 meV with a monolayer h-BN tunneling layer. These SBHs may be underestimated due to the long channel length.^42^ However, through the controlled device structure with and without h-BN layer, the reduced value for the monolayer h-BN/Au contact confirms that h-BN tunneling layer can effectively decrease the SBH.

As the semiconductor contacts the metal, the metal’s wave function penetrates the semiconductor. This penetration induces rehybridization of the semiconductor’s wave function, resulting in the formation of gap states within the semiconductor’s bandgap, as illustrated in Figure 3e.^41^ Introducing a monolayer h-BN tunneling layer reduces metal-induced gap states (MIGS) without altering the MoS_2_ bands, while effectively lowering the SBH by modifying the band alignment at the interface (Figure 3f). For the metal/insulator/semiconductor contact, the contact resistance is determined by the Schottky barrier and tunneling resistance with the latter being relatively small due to the atomic thickness (∼0.42 nm) of the monolayer h-BN.^43^ The most intuitive manifestation of the reduction in contact resistance is the increase in ON-current. Figure 3g shows the transfer curves of back-gated MoS_2_ transistors on SiO_2_ substrates. With the monolayer h-BN, the ON-current of the MoS_2_ transistor shows an increase from 11 μA μm^–1^ to 15 μA μm^–1^. Transfer curves of the MoS_2_ transistors with varying channel lengths are presented in Figure S5. The contact resistance is further extracted by the transmission line method and shown in Figure 3h. The device with the monolayer h-BN contact exhibits a smaller contact resistance of 4.7 kΩ μm compared to 7.1 kΩ μm for the direct Au contact.

### High-Performance 2T0C DRAM Based on Damaged-Free Top-Gate MoS_2_ Transistors

**Figure 4:**
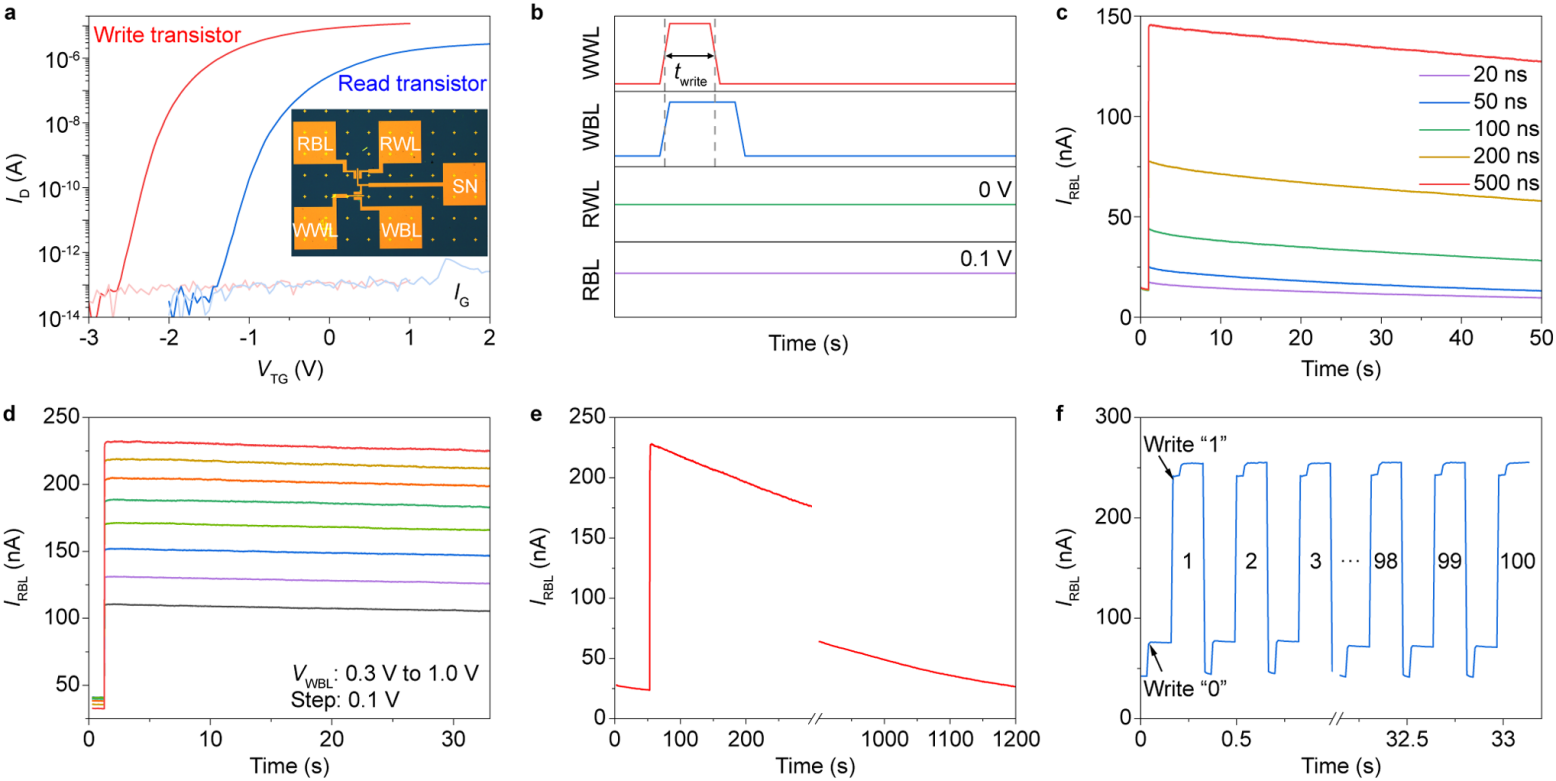
Electrical performances of the 2T0C DRAM. (a) Transfer curves of the write and read MoS_2_ transistors. The inset shows the optical image of the 2T0C DRAM cell with two MoS_2_ transistors. The channel width/length of the write and read transistors are 14 μm/10 μm and 7 μm/10 μm, respectively. (b) Timing diagram of the write and read operations for 2T0C DRAM. (c) *I*_RBL_ as a function of read time following the writing of data “1”, with the write pulse width varying from 20 to 500 ns. The amplitude of the write pulse is fixed to be 1 V. (d) Data retention of 3-bit DRAM with *V*_WBL_ ranging from 0.3 to 1.0 V. (e) *I*_RBL_ evolution of the fabricated 2T0C DRAM, following the writing of data “1”, demonstrates a retention time exceeding 1,000 s. (f) 100 times write and erase operations of data “1”. Each conducted with a pulse width of 60 ms and pulse amplitude of 1 V.

The h-BN-assisted vdW dielectric/electrode transfer method enables the fabrication of transistors with ultralow leakage current, which is crucial for extending DRAM retention time. Utilizing this one-step transfer method, we simultaneously fabricated two damage-free MoS_2_ transistors to construct a 2T0C DRAM cell. Compared to conventional 2T0C DRAM fabrication processes, which require steps such as photolithography, metallization, and etching, this vdW integration method shows greater compatibility with 2D materials (Figure S6).^44^ The insert of Figure 4a presents the optical image of the 2T0C DRAM cell, which comprises a write transistor and a read transistor. In this configuration, the drain of the write transistor is connected to the gate of the read transistor. The gate capacitance of the read transistor is used for charge storage. As illustrated in the circuit diagram in Figure S7, the write and read operations of the 2T0C DRAM cell are separated. The state of the DRAM can be read by monitoring the channel current of the read transistor, thereby ensuring that the stored charge in the gate capacitor remains undisturbed. Figure 4a shows the transfer curves of the two transistors within the 2T0C DRAM cell. The corresponding output curves of the two devices are shown in Figure S8. Both transistors exhibit exceptionally low leakage currents, which is advantageous for prolonging data retention time. It is noteworthy that the MoS_2_ channel of the write transistor is thicker (∼6 nm) than that of the read transistor, to achieve a higher ON state current and thereby enhance the write speed. Conversely, the MoS_2_ channel of the read transistor is designed to be thinner (∼3.5 nm), ensuring operation within the subthreshold region at a gate voltage of 0 V, which optimizes the sense margin. It is important to note that an excessively positive or negative threshold voltage of the read transistor will reduce the sense margin.

Figure 4b illustrates the timing diagrams that delineate the write and read operations for the 2T0C DRAM cell. To continuously monitor the stored charge in the storage node, the read bit line (RBL) and read word line (RWL) were set to 0.1 and 0 V, respectively. During a write “1” operation, the write bit line (WBL) was set to a longer pulse duration than the write word line (WWL) to prevent charge leakage. First, we performed a write speed test on the 2T0C DRAM cell. Following a 20 ns write pulse, the *I*_RBL_ response for data “0” and data “1” exhibits a distinguishable difference, as shown in Figure 4c. The calculated energy consumed during a write operation is approximately 0.2 fJ, indicating that our device can operate with extremely low power consumption. When we extend the write duration time from 20 to 500 ns, the current level of data “1” also increases. This rapid write speed is intrinsically linked to the high ON-current of the write transistor. However, the assessment of nanosecond-level write speed is influenced by RC delay in the interconnects, which may result in a reduction in writing efficiency.

A longer write pulse duration of 10 ms to the DRAM results in a high current level of 230 nA. This large state difference also facilitates multilevel storage capability within a single DRAM cell. By fixing the *V*_WWL_ at 1 V with a write pulse duration of 10 ms and varying the *V*_WBL_ from 0.3 to 1 V in increments of 0.1 V, eight distinct memory states are achieved, as illustrated in Figure 4d. The output current *I*_RBL_ demonstrates eight distinct levels, which remain stable throughout the entire measurement period of 30 s. To evaluate the data retention time of the 2T0C DRAM, *I*_RBL_ over time is recorded after write “1”, as shown in Figure 4e. The sense margin between logic “1” and “0” reaches 200 nA after write operation, attributed to the suitable *V*_TH_ of the read transistor. Following the write “1” operation, the *I*_RBL_ gradually decreases due to the extremely small leakage current of the top-gate MoS_2_ transistors. Nevertheless, even after 1,000 s, data “1” and data “0” remain distinguishable. Based on the observed long data retention of this 2T0C DRAM, the leakage current is calculated to be less than 1.34 × 10^–15^ A. As a storage device, DRAM requires frequent write and erase operations. Therefore, we conducted 100 write and erase cycles, as depicted in Figure 4f. The current levels corresponding to data “1” and data “0” exhibited negligible changes, thereby demonstrating the device’s exceptional stability.

## Conclusion

In conclusion, the h-BN-assisted vdW integration strategy presents a great advancement in the fabrication of damage-free top-gate 2D transistors with pristine interfaces. By employing a monolayer h-BN as a buffer layer, this method effectively separates the high-energy deposition processes from the 2D materials, thereby preserving their intrinsic properties. The resulting MoS_2_ top-gate transistors exhibit remarkable electrical performance, including a high ON/OFF ratio up to 10^8^, small SS of 84 mV dec^–1^, low leakage current of approximately 10^–7^ A cm^–2^, and very low interface trap density of about 3 × 10^11^ cm^–2^ eV^–1^. Additionally, the reduced SBH and lower contact resistance achieved through this approach further enhance device performance. Furthermore, the successful demonstration of a 2T0C DRAM cell with two top-gate MoS_2_ transistors underscores the potential of this technology in improving DRAM performance, particularly in terms of data retention and write speed. These findings highlight the promise of vdW integration techniques in advancing the capabilities of next-generation electronic devices, particularly those utilizing 2D materials.

## Experimental Section

The h-BN (Sixcarbon Technology Shenzhen), grown via chemical vapor deposition (CVD) on copper foil, was transferred onto a sacrificial SiO_2_/Si substrate using a wet transfer method. Subsequently, the sample was annealed under a mixed flow of H_2_/N_2_ at 350 °C for 3 h to release strain and remove any PMMA residue.^45^ The bulk MoS_2_ samples were purchased from HQ Graphene, and few-layer MoS_2_ flakes were mechanically exfoliated onto SiO_2_/n^++^Si substrates.

Photolithography was utilized to define the gate dielectric region, followed by the deposition of an approximately 20 nm Al layer using an electron-beam evaporator at a pressure of 10^–6^ Torr and a deposition rate of 0.3 Å/s. This was followed by a liftoff process. The Al was then annealed in air at 400 °C for 3 h to form a uniform Al_2_O_3_ dielectric layer. Subsequently, 40 nm Au source, gate, and drain electrodes were patterned using photolithography and deposited via an electron-beam evaporator.

To fabricate the device, a PVA film was employed to mechanically peel off the dielectric/electrode stack. The PVA film, along with the dielectric/electrode stack, was affixed to a polydimethylsiloxane (PDMS) stamp, which was attached to a glass slide. Using a 2D materials transfer platform, the dielectric/electrode stack and the MoS_2_ flake were precisely aligned. The dielectric/electrode stack, along with the PVA, were then adhered to the MoS_2_ flake. The devices were heated to 68 °C for 3 min to facilitate the release of the PDMS. The PVA film was subsequently dissolved using deionized water. Finally, the devices underwent annealing in a vacuum at 200 °C for 2 h to enhance the contact quality. The same method was used to fabricate Au/MoS_2_/h-BN/Al_2_O_3_/Au device on quartz substrate for the C–V test.

The cross-sectional sample of the MoS_2_/h-BN/Al_2_O_3_ stack was prepared using a Helios 5 CX DualBeam focused ion beam. STEM characterization and EDS analyses were carried out on a ThermoFisher Scientific Spectra 300 TEM with an accelerating voltage of 300 kV. To determine the roughness of the surface at the interface between Al_2_O_3_ and MoS_2_, the Al_2_O_3_ was peeled off using PVA and subsequently placed on top of PDMS. The surface was then scanned using a Bruker MultiMode 8 scanning probe microscope.

Electrical characterization was performed in a Lakeshore TTPX probe station using a Keithley 4200A SCS parameter analyzer at atmospheric conditions. The 2T0C DRAM measurements were conducted with a Keysight B1500A semiconductor characterization system, while the pulse voltage was applied using the SPGU module.

## Supporting Information Available

XPS of the Al_2_O_3_ film; optical images of the device stacks during the transfer process; output curves of the damage-free MoS_2_ transistor; transfer curves of the MoS_2_ transistors under different temperatures; transfer curves of the MoS_2_ transistors with different channel lengths; DRAM fabrication process; 2T0C DRAM circuit diagram; output curves of the two damage-free MoS_2_ transistors in one 2T0C DRAM cell (PDF)
