# Supplementary material for: Capacitorless Dynamic Random Access Memory with 2D Transistors by One-Step Transfer of van der Waals Dielectrics and Electrodes
Source: ACS Nano. 2025 Jan 10;19(2):2848–56. doi: 10.1021/acsnano.4c15750 (PMC11760144; doi:10.1021/acsnano.4c15750)
Supplement: Supplementary file 1 — nn4c15750_si_001.pdf [file nn4c15750_si_001.pdf]

Supporting Information for

Capacitorless Dynamic Random Access Memory with 2D Transistors by One-Step Transfer of van der Waals Dielectrics and Electrodes

Jianmiao Guo[1,2], Ziyuan Lin[1,2], Xiangli Che[1,3], Cong Wang[1,2], Tianqing Wan[1,2], Jianmin Yan[1,2], Ye Zhu[1,3], and Yang Chai[1,2]*

[1] Department of Applied Physics, The Hong Kong Polytechnic University, Kowloon, Hong Kong 999077, China.

[2] Joint Research Centre of Microelectronics, The Hong Kong Polytechnic University, Kowloon, Hong Kong 999077, China.

[3] Research Institute for Smart Energy, The Hong Kong Polytechnic University, Kowloon, Hong Kong 999077, China.

* Corresponding author: Yang Chai, Email: ychai@polyu.edu.hk

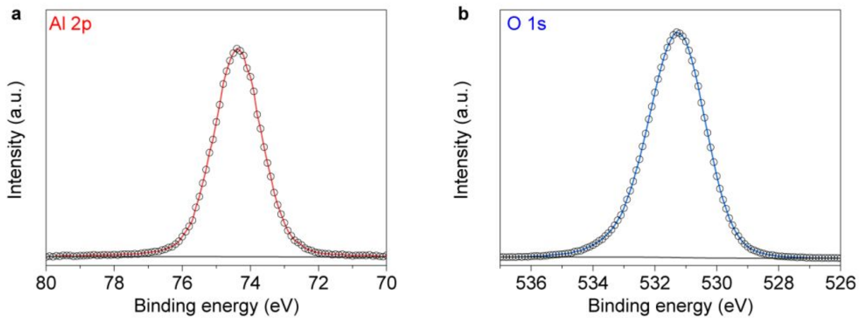

Figure S1. XPS of the Al_2O_3 film. Peak fitting of (a) Al 2p signal and (b) O 1s signal.

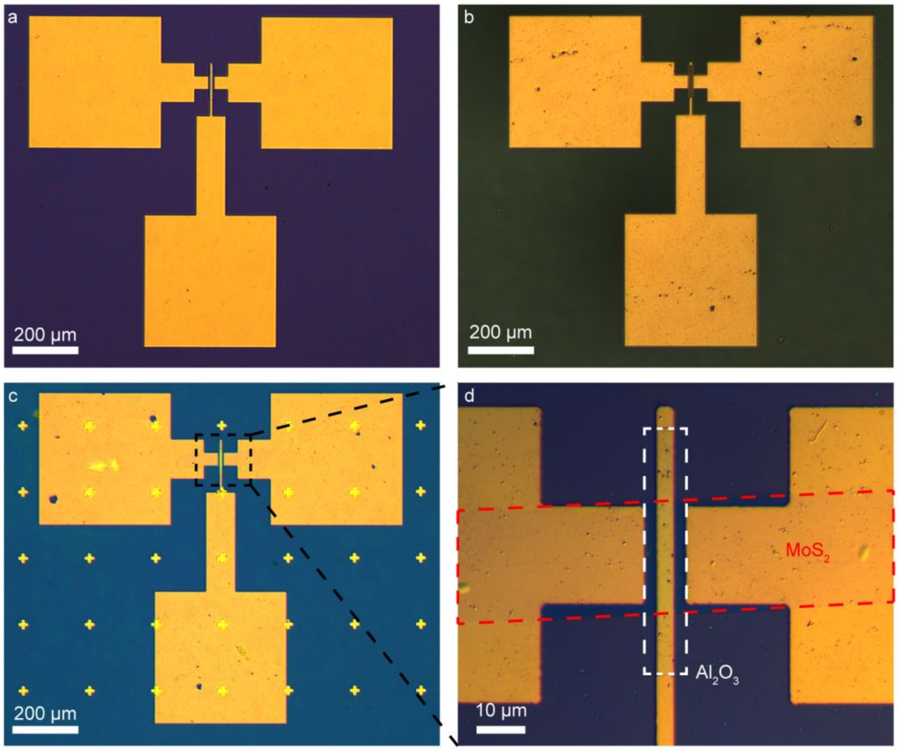

Figure S2. Optical images of the device stacks during the transfer process. (a) Optical microscopy image of S/G/D electrodes and gate dielectric stack on monolayer h-BN on SiO_2 sacrificial substrate. (b) Optical microscopy image of the peeled-off top-gated device stack. (c) Optical microscopy image of the vdW-integrated top-gated MoS2 transistor. (d) Enlarged image of the area marked in (c).

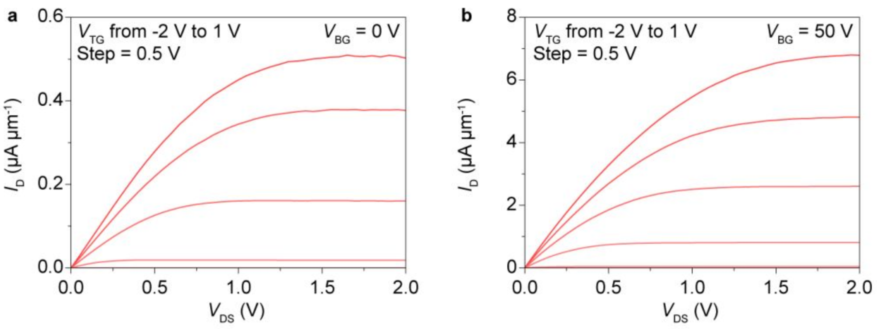

Figure S3. Output curves of the damage-free MoS_2 transistor. (a) Output curves of the MoS_2 transistor under 0 V back-gate voltage. (b) Output curves of the MoS_2 transistor under 50 V back-gate voltage.

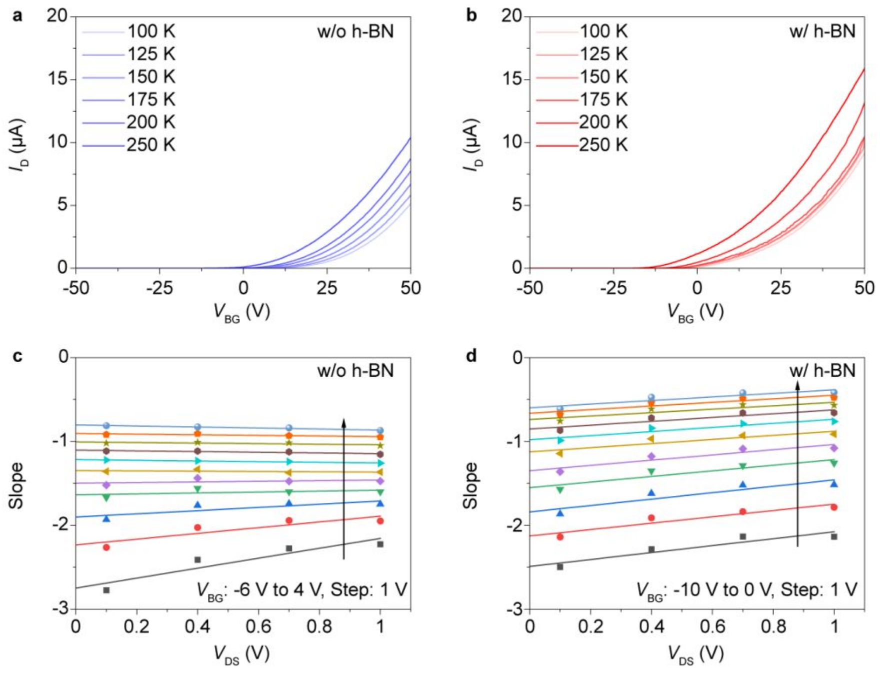

Figure S4. Transfer curves of the MoS_2 transistors under different temperatures and barrier height extraction. (a) Transfer characteristics of MoS_2 transistor with direct Au contact. (b) Transfer characteristics of MoS_2 transistor with monolayer h-BN/Au contact. Channel length = 2 μm, V_{DS} = 1 V. (c) Extracted slope as a function of V_{DS} (direct Au contact). Φ_b is derived from the y-intercept. (d) Extracted slope as a function of V_{DS} (monolayer h-BN/Au contact). Φ_b is derived from the y-intercept.

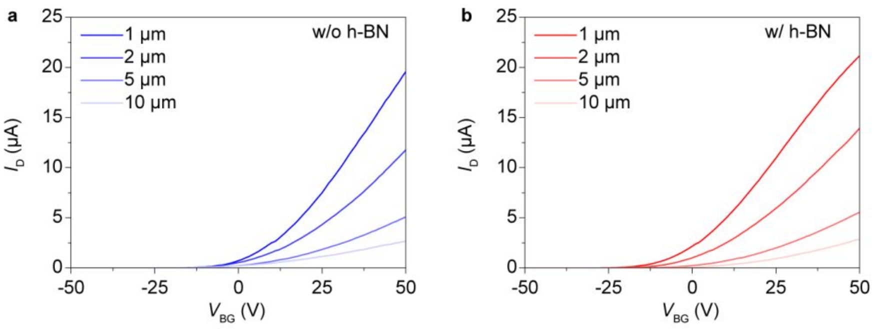

Figure S5. Transfer curves of the MoS_2 transistors with different channel lengths. (a) Transfer curves of MoS_2 transistor with direct Au contact. (b) Transfer curves of MoS_2 transistor with monolayer h-BN/Au contact. $V_{DS} = 1$ V.

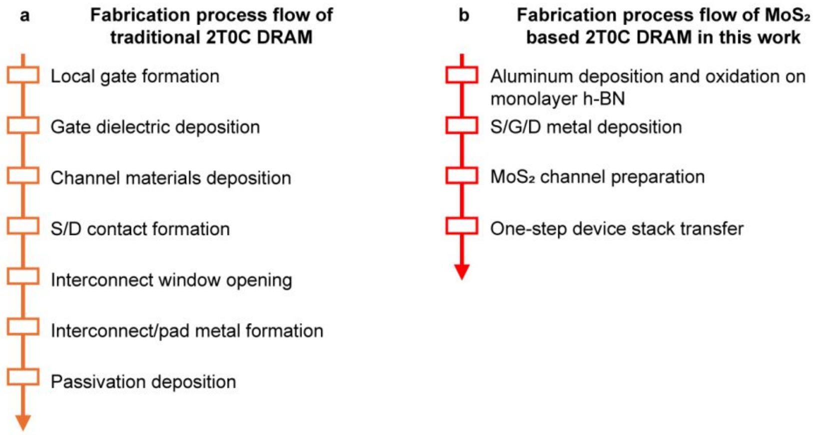

Figure S6. DRAM fabrication process. Fabrication process flow of traditional 2T0C DRAM (a) and MoS_2 based 2T0C DRAM (b).

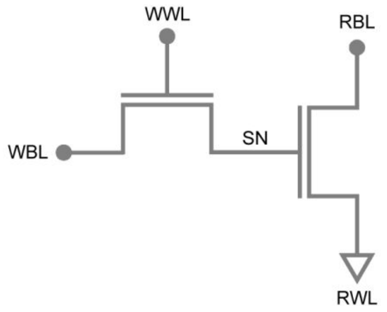

Figure S7. 2T0C DRAM circuit diagram. Circuit diagram of the 2T0C DRAM cell where the data is stored in the gate capacitance of the read MoS_2 transistor. WBL, WWL, RBL, RWL, and SN refer to write bit line, write world line, read bit line, read world line, and storage node respectively.

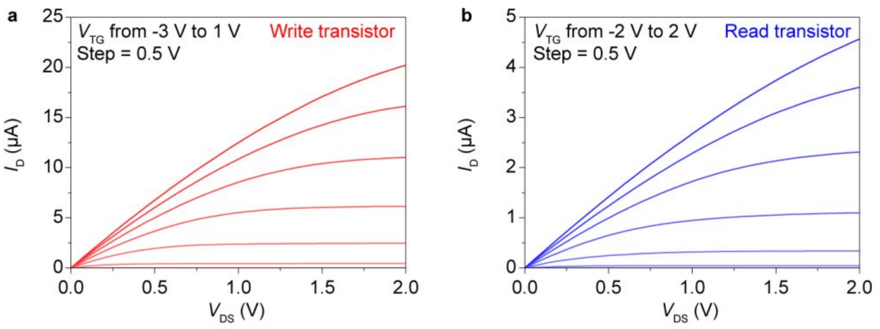

Figure S8. Output curves of the two damage-free MoS_2 transistors in one 2T0C DRAM cell. (a) Output curves of the write MoS_2 transistor. (b) Output curves of the read MoS_2 transistor.
